# Transylvania Medical Journal

**Published:** 1849-10

**Authors:** 


### TRANSYLVANIA MEDICAL JOURNAL.

A new periodical, with the above title, under the auspices of the Medical Faculty of Transylvania University, and edited by Professor Ethelbert L. Dudley, has been commenced at Lexington, and is to be published in bi-monthly numbers. We cordially welcome our young cotemporary into the field and wish it a long and prosperous career. Twenty-two years ago a predecessor, bearing nearly the same name, was ushered to public notice under auspices similar to those which attend the present enterprise. Under various editors, but with unvarying fortunes, it struggled hard against the tide for years, and was finally discontinued. What was its fate has been the fate of nearly every journal that has succeeded it on our side of the mountains. At first, contributors write and subscribers pay with encouraging punctually; but anon the novelty wears off; the hippocrene of many a writer runs dry, and the "patrons" forget that publishers and printers are men, and like other mortals must be clothed and fed. We hope such will not be the fate of the young "phenix," at Lexington, but that the spirit with which it starts upon its course will be maintained and improved. The first number contains several articles of interest, and its whole appearance is promising. If we were in a captious humor there are some things in it which we might find occasion to criticize; but we prefer to pass these over in silence and to give the new candidate for public favor a friendly greeting on its first appearance upon the stage.

